# In vivo assessment of OXPHOS capacity using 3 T CrCEST MRI in Friedreich’s ataxia

**DOI:** 10.1007/s00415-021-10821-1

**Published:** 2021-10-15

**Authors:** Gayatri Maria Schur, Julia Dunn, Sara Nguyen, Anna Dedio, Kristin Wade, Jaclyn Tamaroff, Nithya Mitta, Neil Wilson, Ravinder Reddy, David R. Lynch, Shana E. McCormack

**Affiliations:** 1 Division of Endocrinology and Diabetes, The Children’s Hospital of Philadelphia, Philadelphia, PA 19104, USA; 2 Medical Scientist Training Program, New York University Grossman School of Medicine, Vilcek Institute of Graduate Biomedical Sciences, 550 First Avenue, MSB 228, New York, NY 10016, USA; 3 Center for Magnetic Resonance and Optical Imaging, Department of Radiology, University of Pennsylvania, Philadelphia, PA 19104, USA; 4 Division of Neurology, The Children’s Hospital of Philadelphia, Philadelphia, PA 19104, USA; 5 Department of Pediatrics, Perelman School of Medicine, University of Pennsylvania, Philadelphia, PA 19104, USA; 6 Department of Neurology, Perelman School of Medicine, University of Pennsylvania, Philadelphia, PA 19104, USA

**Keywords:** Friedreich’s ataxia, Magnetic resonance imaging, Skeletal muscle, OXPHOS, Oxidative metabolism, Exercise, Mitochondrial disorders

## Abstract

**Background:**

Friedreich’s ataxia (FRDA) is a neurodegenerative disease caused by decreased expression of frataxin, a protein involved in many cellular metabolic processes, including mitochondrial oxidative phosphorylation (OXPHOS). Our objective was to assess skeletal muscle oxidative metabolism in vivo in adults with FRDA as compared to adults without FRDA using chemical exchange saturation transfer (CrCEST) MRI, which measures free creatine (Cr) over time following an in-magnet plantar flexion exercise.

**Methods:**

Participants included adults with FRDA (*n* = 11) and healthy adults (*n* = 25). All underwent 3-Tesla CrCEST MRI of the calf before and after in-scanner plantar flexion exercise. Participants also underwent whole-body dual-energy X-ray absorptiometry (DXA) scans to measure body composition and completed questionnaires to assess physical activity.

**Results:**

We found prolonged post-exercise exponential decline in CrCEST (τCr) in the lateral gastrocnemius (LG, 274 s vs. 138 s, *p* = 0.01) in adults with FRDA (vs. healthy adults), likely reflecting decreased OXPHOS capacity. Adults with FRDA (vs. healthy adults) also engaged different muscle groups during exercise, as indicated by muscle group-specific changes in creatine with exercise (ΔCrCEST), possibly reflecting decreased coordination. Across all participants, increased adiposity and decreased usual physical activity were associated with smaller ΔCrCEST.

**Conclusion:**

In FRDA, CrCEST MRI may be a useful biomarker of muscle-group-specific decline in OXPHOS capacity that can be leveraged to track within-participant changes over time. Appropriate participant selection and further optimization of the exercise stimulus will enhance the utility of this technique.

## Introduction

Friedreich’s ataxia (FRDA) is a rare neurodegenerative disease caused by GAA triplet-repeat expansions in the *FXN* gene, which encodes the protein frataxin [1]. Frataxin regulates the assembly of iron–sulfur clusters necessary for mitochondrial oxidative phosphorylation (OXPHOS) [2]. The GAA triplet-repeat expansion leads to decreased *FXN* expression and therefore decreased frataxin levels and impaired mitochondrial OXPHOS capacity. Individuals with FRDA have impaired skeletal muscle OXPHOS capacity as measured by ^31^P magnetic resonance spectroscopy (MRS), which has historically been used to assess OXPHOS capacity in vivo by measuring changes in phosphocreatine (PCr) over time in response to exercise [3, 4]. However, the limited spatial resolution of ^31^P-MRS makes assessment of muscle-group-specific OXPHOS capacity challenging. This is a significant disadvantage of ^31^P-MRS because different muscle groups may differ in myofiber type composition, and different myofiber types have distinct metabolic properties and mitochondrial OXPHOS capacity [5].

Chemical exchange saturation transfer (CEST) MRI is a contrast enhancement technique that can indirectly measure endogenous metabolites through their exchange with bulk water [6, 7]. When saturation pulses are applied to the amine protons of creatine, this saturated magnetization exchanges with bulk water protons in the surrounding tissue, resulting in a decrease in water signal proportional to Cr concentration. Using this creatine-weighted CEST contrast (CrCEST), free creatine (Cr) can be measured over time in response to a provocative exercise stimulus to indirectly assess skeletal muscle oxidative metabolism [8]. As compared to ^31^P-MRS, CrCEST MRI has the added benefit of enhanced sensitivity that allows for high spatial resolution imaging, making muscle group-specific assessment of metabolism possible. In addition, CrCEST can be performed with a standard ^1^H coil, while ^31^P-MRS requires multinuclear hardware not as widely available [6].

Recently, our group detected differences in OXPHOS capacity in the medial gastrocnemius muscle between adults with diverse primary mitochondrial disorders (mostly non-FA) and healthy adults using CrCEST at 7 T [9]. In this study, we used the CrCEST technique to assess skeletal muscle OXPHOS capacity in individuals with FRDA relative to individuals without FRDA at 3 T, a more widely available field strength. We aimed to characterize the relationship between OXPHOS capacity and candidate clinical factors expected to impact muscle metabolism, including body composition and physical activity.

## Materials and methods

### Study design and participants.

This study was conducted under an approved Institutional Review Board protocol. Written informed consent for each participant was obtained. Adults (ages 18–65 years) with a confirmed genetic diagnosis of FRDA, without diabetes mellitus, were recruited for a cross-sectional, observational metabolic phenotyping study (NCT02920671) that included CrCEST imaging. Healthy adults without diabetes mellitus were recruited to generate a control cohort with a similar distribution of age, sex, body mass index (BMI), and population ancestry as individuals with FRDA.

### Waist circumference and body composition.

Waist circumference was measured at the upper aspect of the iliac crest. Body composition (whole body and regional) was assessed using the Horizon A Platform (Hologic, Inc., Bedford MA) with Apex software v5.5 at the Children’s Hospital of Philadelphia Growth and Nutrition Laboratory. Outcomes assessed included whole-body lean and fat mass, and leg lean and fat mass.

### Physical activity.

Adults completed the Chronic Renal Insufficiency Cohort (CRIC) Physical Activity Questionnaire to generate estimates of usual physical activity, including time spent in light-, moderate-, and heavy-intensity exercise, intentional exercise, and total physical activity [10].

### Magnetic resonance imaging.

CrCEST imaging was performed using a 15-channel ^1^H Tx/Rx knee coil (Quality Electrodynamics, Mayfield Village, OH, USA) on a 3 T whole-body scanner (MAGNETOM Prisma, Siemens Healthcare, Erlangen, Germany) at the University of Pennsylvania (Philadelphia, PA). CrCEST imaging parameters were as follows: TR/TE = 4.7/2.3 ms, slice thickness = 10 mm, flip angle = 10°, saturation B1_rms_ = 3 μT, saturation duration = 500 ms, in plane resolution = 1.25 × 1.25 mm^2^, matrix size = 128 × 128, field of view = 160 × 160 mm^2^. Time between saturation was 4 s, and offsets were acquired at /pm 1.5, 1.8, and 2.1 ppm relative to water. Water saturation shift reference (WASSR) and B _1_^+^ maps were acquired, to correct for B0 and B1 field inhomogeneities [11, 12].

The same exercise-based imaging procedures were as follows. First, five baseline images of the right calf were acquired with a temporal resolution of 24 s. Then, participants performed a plantar flexion exercise for 2 min inside the scanner using an MR-compatible ergometer (Penn), held at a fixed resistance. The individualized exercise stimulus was designed to be mild; prior to scanning, capacity to perform the exercise was assessed. In healthy adult volunteers, 12 psi was used. In individuals with FRDA with limited exercise capacity, resistance was decreased to either 8 or 4 psi, as appropriate, if participants could not easily depress the pedal with the 12-psi resistance. All participants were coached to complete the exercise at a frequency of 45 repetitions per minute, with adherence verified visually. Participants with limited capacity to perform the exercise were directed to keep cadence to the extent possible. The goal of the exercise stimulus was to deplete PCr and increase Cr sufficiently to measure post-exercise Cr decline. Eight minutes of post-exercise images were acquired at the same temporal resolution of 24 s.

### Image processing.

Image processing was performed using an in-house written MATLAB (version 2019a) program [13]. CEST contrast (MTR_asym_) is computed via z-spectra asymmetry analysis [6]. To determine MTR_asym_ for individual muscle groups, anatomical images were manually segmented for the muscles most involved in the plantar flexion movement, which included the lateral gastrocnemius (LG), medial gastrocnemius (MG), and soleus (Sol) muscles in the calf, and these segmentations were applied to the CrCEST maps. MTR_asym_ of the voxels included in each segmented muscle group were then averaged for each timepoint. For a given muscle, resting CrCEST (prior to exercise) was computed as the average CrCEST from the baseline images, representing the free creatine concentration at rest. Change in CrCEST with exercise (ΔCrCEST) was computed as the change between resting CrCEST and the value of MTR_asym_ at the first post-exercise timepoint, representing the change in free creatine with exercise. According to the kinetics of the phosphocreatine (PCr) shuttle, decline in free creatine concentration after exercise is typically modelled as an exponential function [8]. Thus, the post-exercise exponential decline in CrCEST (τCr) is the main outcome reflecting OXPHOS capacity. CrCEST imaging has sufficiently high anatomic resolution to perform muscle-group-specific analyses; thus, τCr was computed by fitting muscle-specific MTR_asym_ timeseries to an exponential model.

Based on our previous experience, physiologically relevant values of τCr are expected to fall between ~ 50 and 300 s [9]. τCr estimates less than one inter-scan interval (24 s) were excluded because of the limited temporal resolution. Values of τCr greater than 1,000 s, well above the upper range of the τCr distribution (over all measurements, the 95th percentile in healthy controls was 399 s and in individuals with FA was 449 s) and beyond the post-exercise scan duration (480 s), were also excluded as implausible and likely due to an insufficient exercise response.

### Statistical analyses.

The Shapiro–Wilk test was used to test the normality of distribution for CrCEST imaging outcomes. Kruskal–Wallis tests were performed in non-normally distributed outcome measures. Linear mixed-effects models were used to assess the effect of disease status on each muscle-group-specific CrCEST imaging outcome, adjusting statistically for clinical covariates, while also accounting for subject-specific random effects. For the outcomes assessed, if an interaction effect between disease status and muscle group was detected, suggesting that the impact of disease status on the CrCEST outcome varied between muscle groups, then linear regression models were built for each muscle group (LG, MG, Sol) separately to investigate the effect of covariates. In all models, subject is included as a random effect and sex, age, and disease status were included as fixed effects. Sex and age were included because skeletal muscle composition varies with these clinical characteristics.

Statistical models testing the outcome of resting CrCEST included age, sex, disease status, muscle group, and the following additional fixed effects: no additional effects (Model 1); BMI (Model 2); height, and right leg lean tissue mass (Model 3); height and right leg fat mass (Model 4). Statistical models testing the outcome of ΔCrCEST included age, sex, disease status, and the following additional covariates: no additional covariates (Model 1); total physical activity (Model 2); waist circumference (Model 3). Statistical models testing the outcome of τCr included sex, age, disease status and the following additional covariates: no additional covariates (Model 1); ΔCrCEST and resting CrCEST (Model 2); total physical activity (Model 3). Statistical interactions between disease status and muscle group were detected in mixed-effects models of both ΔCrCEST and τCr, so linear regression models of each outcome were built for each muscle group separately. All statistical analyses were done in RStudio (Version 1.1.383), and statistical significance was taken to be a two-sided *p* value of < 0.05.

## Results

### Participants.

Characteristics of study participants are summarized in Table 1. By design, there were similar proportions of individuals who were obese between FRDA and control cohorts, and a nominally higher proportion of individuals with FRDA were in the overweight relative to normal weight category as compared to controls. Fifty-five percent of individuals with FRDA and 40% of controls had increased waist circumference for age, defined as > 88 cm for females and > 102 cm for males [14]. Individuals with FRDA reported spending less time in total weekly physical activity, as well as less time in light- and heavy-intensity exercise as compared to control participants.

## CrCEST imaging

**Fig. 1:**
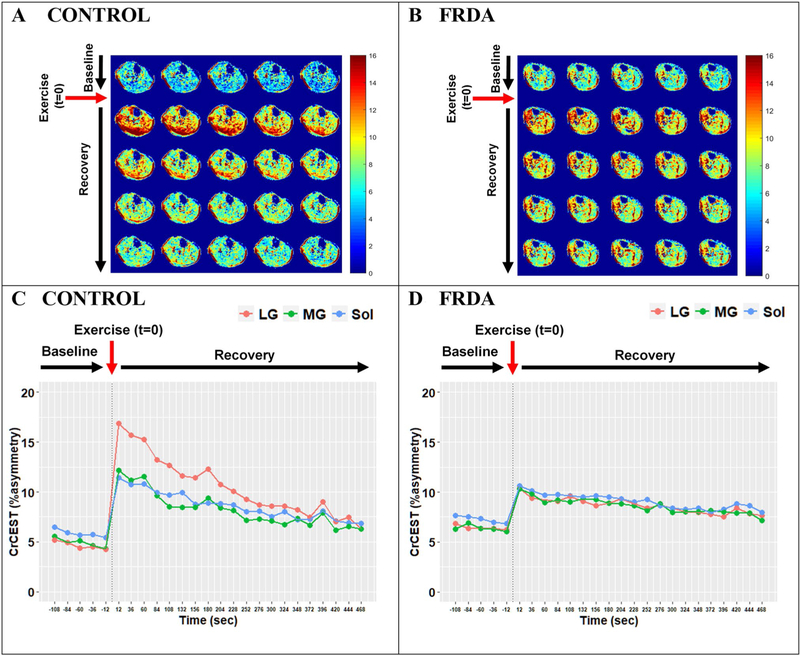
CrCEST over time for a healthy 25yo male and a 30yo male with FRDA, respectively. (**A**, **B**) Maps of CrCEST (%asymmetry), rest–exercise–recovery protocol for (**A**) healthy 25yo male where τCr = 269 s, 263 s, 287 s and (**B**) 30yo male with FRDA where τCr = 741 s, 451 s, 367 s for the LG, MG, and soleus, respectively. The color bar indicates the intensity of the CrCEST signal, in proportion to the concentration of Cr in the muscle. (**C, D**) CrCEST over time for the same participants; prolonged recovery corresponds to decreased OXPHOS capacity. CrCEST in the LG at *t* = 60 s was omitted from the time series plot in Fig. 1D due to an error in image acquisition at that timepoint but was included in model fitting. LG: lateral gastrocnemius, MG: medial gastrocnemius, Sol: soleus

CrCEST imaging outcomes (resting CrCEST, ΔCrCEST following exercise, and τCr) are summarized in Table 2. Individual CrCEST maps and corresponding time series are shown for two representative participants in Fig. 1, including a healthy 25yo male and a 30yo male with FRDA. Three scans (1 case and 2 controls) were excluded due to data collection limitations, including significant motion artifacts and field inhomogeneity correction errors. Two observations of τCr in the MG were less than one scan time (24 s) and were, therefore, excluded. Otherwise, other fitted values fell within the specified plausible range of 24–1,000 s and were therefore included in the analysis.

### Resting CrCEST.

In univariate analyses, we did not detect a difference between FRDA and controls in resting CrCEST, an index of free creatine concentration at rest (Table 2, Supplementary Fig. 1). We also did not detect the presence of any muscle group-specific interaction effects with disease status, so we investigated the effects of other potentially relevant covariates on resting CrCEST analyzing all muscle groups together. Independent of disease status, resting CrCEST was higher in the soleus as compared to the LG in all models (*p* = 0.002) (Table 3). Also independent of disease status, higher BMI (β = −0.11, *p* = 0.006) was associated with lower resting CrCEST (Table 3, Model 2). To better understand the relationship between BMI and resting CrCEST, we examined models including more detailed body composition measures from DXA scans. Both lean mass (β = −0.42, *p* = 0.025) and fat mass (β = −0.32, *p* = 0.007) in the right leg were also associated with lower resting CrCEST in adults when accounting statistically for age, sex, and height. Because larger individuals tend to have more fat mass as well as more lean mass, we tested a model including both lean and fat mass in the leg, but likely due to the sample size, we were unable to discern which of these contributed most to resting CrCEST. Of note, resting CrCEST remained higher in the soleus compared to the LG when accounting for leg lean and fat mass. With respect to other covariates, in our previous study of adults with mitochondrial disease, we detected a positive association between intentional exercise and resting CrCEST in the soleus, but we did not detect an association between physical activity and resting CrCEST in FRDA in this study.^9^

### ΔCrCEST.

In univariate analyses, we did not detect differences between FRDA and controls in ΔCrCEST, a measure reflecting change in free creatine with exercise, in any muscle group (Table 2, Supplementary Fig. 2). This is by design, since our priority outcome measure is post-exercise exponential decline in CrCEST or τCr, which reflects OXPHOS capacity, and we sought to individualize exercise resistance to produce provocative changes in CrCEST with exercise of approximately similar magnitude between cases and controls. However, mixed-effects regression analyses showed a significant interaction effect between disease status and muscle group on exercise ΔCrCEST in adults (*p* < 0.001 for difference between LG and Sol), indicating that individuals with FRDA had different patterns of muscle use with exercise as compared to controls. Specifically, ΔCrCEST with exercise was nominally smaller in the LG and MG in FRDA versus in controls, but ΔCrCEST with exercise in soleus was similar. (Supplementary Fig. 2). This can also be visually observed in individual CrCEST maps, including in Fig. 1, where the control predominantly engages the LG, while the participant with FRDA has a more uniform response across the posterior lower leg.

We next assessed the effects of several clinical covariates on ΔCrCEST within each muscle group using linear regression models (Table 4, Supplementary Table 1). In our linear models, self-reported time spent in total weekly physical activity (MET-hours/week) was associated with a greater ΔCrCEST in the LG (β = 0.18, *p* = 0.02), independent of disease status, sex and age (Table 4). We also found that higher waist circumference (cm) was associated with smaller ΔCrCEST in the MG (β = − 0.06, *p* = 0.005) and soleus muscles (β = − 0.06, *p* = 0.008) across both cases and controls (Supplementary Table 1); a similar pattern was observed in LG, but this result did not reach statistical significance.

### Post-exercise τCr.

**Fig. 2:**
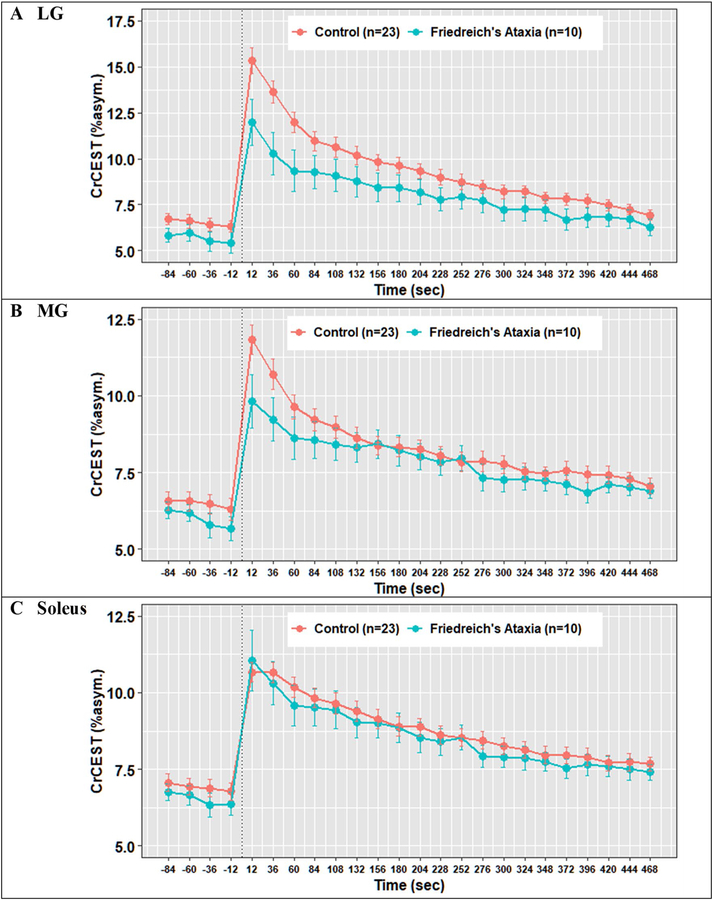
Mean CrCEST timeseries for all adults in the (**A**) lateral gastrocnemius, (**B**) medial gastrocnemius, and (**C**) soleus. In the LG, median τCr = 274 s (IQI 221–309 s) in FRDA and τCr = 138 s (IQI 85–226 s) in controls (p = 0.01 for difference by Kruskal–Wallis test, Table 2)

**Fig. 3:**
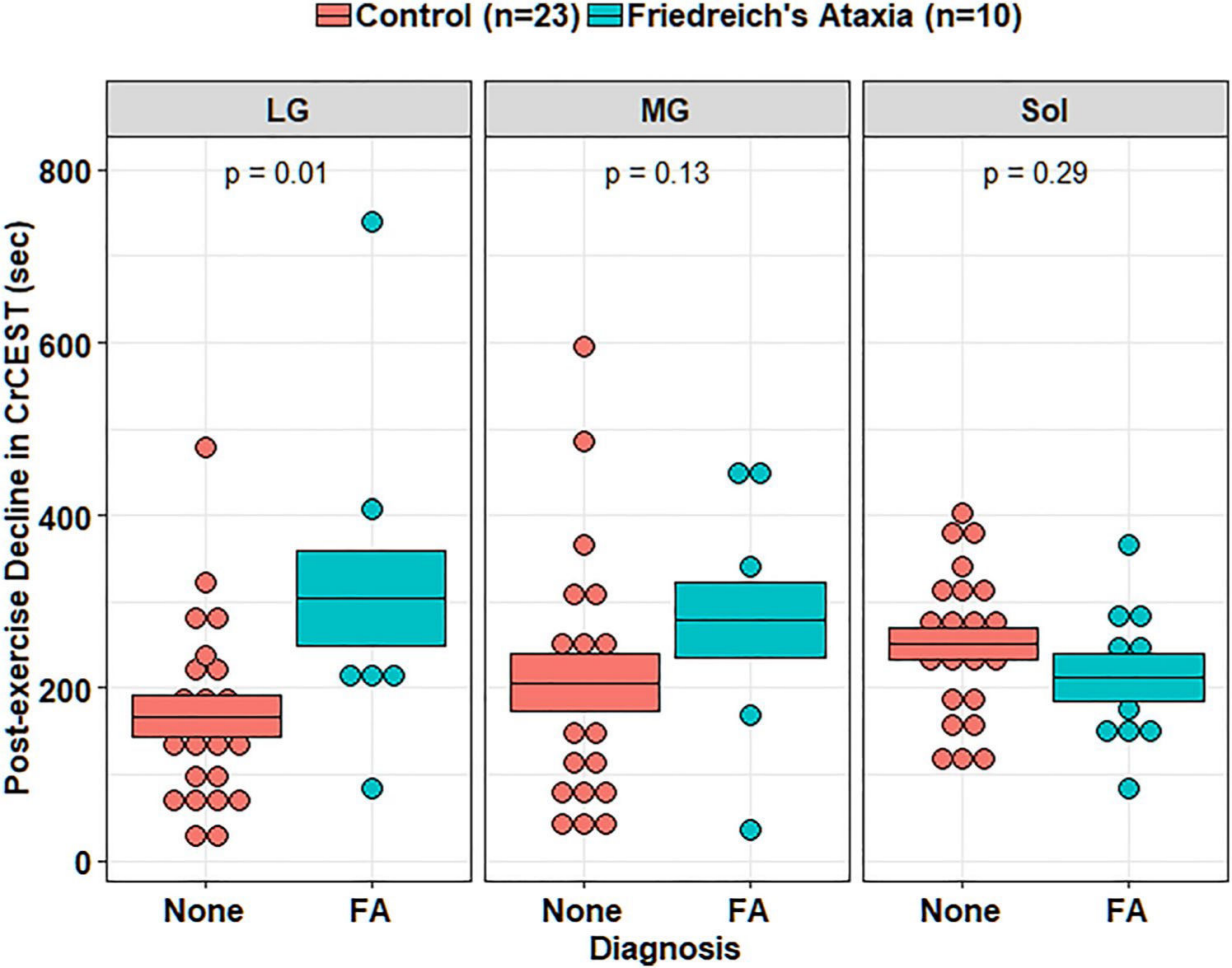
Post-exercise τCr in adults. Orange corresponds to controls, and blue corresponds to adults with FRDA. Median τCr in the LG was 138 s (IQI = 85–227 s) in controls and 274 s (IQI = 221–309 s) in FRDA (Table 2). In a linear regression analysis, FRDA disease status prolongs τCr by 131 s (95% CI = 28–234 s) in the LG, accounting for sex and age (*p* = 0.01, Table 3). This boxplot also illustrates the interaction between disease status and muscle group described, namely that disease-specific differences in post-exercise τCr in adults with FRDA are distinct in soleus as compared to LG. LG: lateral gastrocnemius, MG: medial gastrocnemius, Sol: soleus

In univariate analyses, individuals with FRDA had a prolonged τCr in the LG relative to individuals without FRDA (274 s vs. 138 s, *p* = 0.01), suggestive of decreased OXPHOS capacity in this muscle group (Table 2). In the MG, τCr was 262 s vs. 171 s (nominally longer, but not statistically different), and in soleus 210 s vs. 254 s (similar), in individuals with FRDA and controls, respectively. CrCEST time series for all three muscle groups, averaged for FRDA vs. control, are illustrated in Fig. 2. Dot plots for post-exercise τCr across all three muscle groups are shown in Fig. 3.

In mixed-effects regression analyses of τCr, an interaction effect between disease status and muscle group was detected (*p* < 0.001 for the difference between LG and Sol). The presence of this interaction indicates that the impact of disease status on τCr varies by muscle group, and here, means that the disease-specific differences in post-exercise τCr in LG were not detected in the Sol. We next investigated the effects of potentially relevant clinical covariates including age, sex, BMI, and self-reported physical activity, and technical covariates including resting CrCEST and ΔCrCEST, in successive regression models to test whether the effect of FRDA on post-exercise τCr we observed may be explained by FRDA-related differences in these covariates (Table 5, Supplementary Table 2).

Linear regression models showed a significant effect of FRDA on τCr in the LG in all models including the technical covariates resting CrCEST and ΔCrCEST, thus these covariates did not explain the effect of disease on τCr (Table 5). Model-derived estimates indicate that τCr is prolonged in the LG by 131 s (*p* = 0.01, 95% CI = 28–234 s) in FRDA relative to unaffected individuals, when accounting statistically for sex and age. As a point of reference, median τCr in the LG was 138 s (IQI = 85–227 s) in controls and 274 s (IQI = 221–309 s) in FRDA. Previously, in our study of adults with mitochondrial disease, we detected a correlation between self-reported time spent weekly in intentional exercise and post-exercise τCr [9]. In this study, we detected an association between self-reported physical activity and post-exercise τCr in the MG only, independent of disease status (Supplementary Table 2). Further, the effect of disease status on τCr in the LG became less apparent when accounting for total physical activity, suggesting that physical activity differences may partially explain the effect of FRDA of on τCr.

## Discussion

The main goal of this study was to test a novel metabolic imaging technique, CrCEST MRI, in individuals with FRDA. τCr, an index of skeletal muscle OXPHOS capacity, was prolonged in the LG of adults with FRDA as compared to healthy volunteers, consistent with prior observations that FRDA is associated with decreased skeletal muscle OXPHOS capacity. The association between FRDA and prolonged τCr was persistent (Table 7) after accounting potentially relevant clinical covariates, including age and sex, and experimental factors including ΔCrCEST, an index of relative MRI exercise stimulus intensity, and resting CrCEST, an index of free creatine concentration, in statistical models. However, the impact of FRDA on τCr was attenuated after accounting for total weekly physical activity, suggesting that inactivity may contribute to decreased skeletal muscle OXPHOS capacity in this condition.

Our findings are consistent with previous reports of decreased skeletal muscle OXPHOS capacity in individuals with FRDA obtained using ^31^P-MRS, another MR-based technique that evaluates phosphocreatine metabolism, but whose anatomic resolution is generally more limited than CrCEST MRI [3, 15–17]. Previous ^31^P-MRS reports did not include muscle-group-specific estimates. Notably, we found a statistical interaction between muscle group and the effect of disease status on τCr, indicating that the effect of disease status on OXPHOS capacity differs by muscle group. This finding highlights the utility of the muscle group-specific CrCEST technique. One potential explanation for muscle group differences in FRDA is that muscle group fiber composition may change as a result of disease. Muscle fiber type switching occurs in diverse metabolic conditions, including muscular dystrophies, type 2 diabetes, obesity [18–20]. At least one study has found changes in fiber type composition in both skeletal and cardiac muscle in a mouse model of FRDA; thus fiber type differences could help explain the muscle group differences we see [21].

Several of our observations also suggest that despite our efforts to personalize and adapt the exercise stimulus, individuals with FRDA performed the exercise testing differently than did healthy controls. First, we found a statistical interaction between disease status and muscle group on ΔCrCEST with exercise, indicating that exercise-related muscle group engagement differs in individuals with FRDA as compared to healthy controls. This difference in muscle engagement is also shown in Supplementary Fig. 2. Additionally, when we visually inspected the CrCEST maps, we observed that individuals with FRDA tended to engage all three muscle groups similarly, while healthy controls tended to use the gastrocnemius the most (e.g., Fig. 1). In the context of a plantar flexion exercise, individuals with FRDA with limited coordination and/ or strength may engage all muscle groups, while healthy individuals can efficiently perform the exercise using predominantly the most appropriately positioned muscle group(s). Of note, while we did not include anterior lower leg muscles (e.g., tibialis anterior) in this analysis since they are associated with the dorsiflexion movement, some participants with FRDA can be seen exercising anterior leg muscles, including the individual in Fig. 1B. Engagement of the anterior muscles may be another marker of poor muscle coordination, which occurs in FRDA and likely adversely impacts muscle synergy when performing the plantar flexion movement [1, 22]. Individuals with FRDA may also be weaker, either due to underlying disease and/or deconditioning; indeed, we demonstrated that more self-reported habitual exercise is associated with larger ΔCrCEST with plantar flexion.

Waist circumference and self-reported physical activity were also associated with muscle group-specific exercise parameters, independent of FRDA disease status (Table 4, Supplementary Table 1). Across all participants, more time spent in physical activity was associated with shorter τCr in the MG, suggesting that physical activity is associated with increased OXPHOS capacity in this muscle group. Also, more time spent in physical activity was associated with larger ΔCrCEST with exercise in the LG, indicating that ΔCrCEST may be an index of exercise performance corresponding to increased capacity to depress a pedal in response to individualized resistance. Overall, healthy volunteers also reported more total physical activity (as well as more time spent light- and heavy-intensity exercise) than individuals with FRDA. Exercise intolerance is a common symptom in FRDA and in genetic primary mitochondrial disorders [23–25], and exercise therapy is commonly recommended for patients with mitochondrial disease [26]. Future studies including more detailed assessments of exercise capacity and usual physical activity could add to our understanding. Although there is anecdotal evidence that exercise may be therapeutic in FRDA, the potential benefits of exercise in FRDA for muscle remain the focus of investigation [27]. Another index of adverse cardiometabolic health, larger waist circumference, was associated with smaller ΔCrCEST in the MG and soleus in the entire cohort [28, 29]. Overall, these results demonstrate that physical activity and body composition have meaningful associations with muscle metabolic parameters and may explain some disease-specific effects.

Across all participants, resting CrCEST was higher in soleus as compared to LG, even after accounting for a variety of potentially relevant covariates [30]. Higher resting CrCEST in the soleus (relative to the LG) is consistent with our group’s previous study assessing OXPHOS in healthy individuals and individuals with diverse mitochondrial diseases [9]. Our findings suggest that the soleus has a higher concentration of free creatine relative to the LG, which may be because the soleus muscle is generally observed to have a higher density of slow twitch, oxidative myofibers [31, 32]. Fast twitch fibers have greater PCr content at rest, so our finding may reflect differences in total creatine and/or creatine metabolism between muscle groups with different fiber types [31]. These findings also demonstrate the utility of measuring free creatine, which is not typically measured in muscle energetics studies, and may provide insight into other processes, such as alterations in creatine transport, that could underlie disease-related metabolic deficits. Also, independent of disease status, resting CrCEST was negatively associated with BMI, even after accounting statistically for sex and age (Table 3). We speculate that excess adiposity in over-weight/obese individuals may lead to excess accumulation of intramuscular fat and effective displacement of Cr. We tested a separate statistical model including both right leg fat mass and lean mass in an attempt to dissociate the effects of high fat mass versus high lean mass, since individuals with high BMI tend to have higher amounts of both tissue types, but our model lacked sufficient statistical power to discern independent effects.

This study had several limitations. First, even the careful steps we took to individualize the exercise stimulus did not prevent cohort specific differences in the muscle group pattern of ΔCrCEST with exercise. Since our priority measure in this study is τCr, it would have been optimal to use an exercise stimulus that produced a more similar ΔCrCEST pattern in FRDA versus controls. However, we speculate that inherent disease-specific differences in muscle coordination led to different patterns of muscle engagement that are difficult to eliminate. As previously mentioned, we also did not include anterior lower leg muscles in our analysis, which may provide further insight into muscle coordination in FRDA. With respect to the exercise, the ergometer is calibrated for participants to perform a mild exercise, in part because exercise-induced changes in pH may have effects on CEST signal that are not yet well understood. Thus, another limitation of this study is that we did not measure changes in pH exercise to ensure that in individuals with FRDA, mild exercise does not significantly change pH. Although in our previous study of adults with mitochondrial diseases, pH changes were measured and found to be small with this exercise paradigm [9]. Ultimately, within-participant comparisons of τCr over time with consistent provocative exercise stimuli may be more relevant than differences between individuals with FRDA and controls, between whom the exercise stimulus may be challenging to standardize [33]. Nevertheless, work in this area is ongoing in adults as well as in children, in whom further adaptations to the exercise stimulus are needed. Another technical limitation is the reproducibility of manual segmentation of the muscle groups. Our group previously assessed the inter-observer reproducibility of muscle segmentation in CrCEST, and the mean coefficient of variation for tracing the LG was 14.7% [34]. Lastly, our sample size in this rare disorder limited us from constructing larger statistical models to examine the potential independent contributions of multiple other relevant clinical covariates.

In conclusion, this study had several key findings. We found that individuals with FRDA had a prolonged τCr compared to controls in the LG, suggestive of decreased OXPHOS capacity, even after accounting statistically for technical factors, including differences in how exercise was performed. However, after accounting for differences in physical activity between cohorts, differences in τCr were attenuated, highlighting challenges of detecting the differences between primary (i.e., genetic) and secondary (i.e., related to decreased mobility) adverse effects on muscle metabolism. Optimization of technical factors, including further individualization of the exercise stimulus, may improve the feasibility and utility of CrCEST imaging for noninvasive assessment of OXPHOS capacity. Ultimately, within-individual comparisons over time in τCr may be most informative. We envision continued development of this metabolic imaging tool.

## Supplementary Material

Supplementary material

## Figures and Tables

**Table 1:** Characteristics of adult subjects (healthy volunteers and FRDA)

Characteristic	Healthy volunteers (*n* = 25)	Friedreich’s ataxia (*n* = 11)

Sex (%female, *n*)	44% (11)	36% (4)
Age (years)	29 (25–39)	27 (23–39)
GAA repeats (bp)	NA	633 (467–741)
*Anthropometrics and body composition*		
BMI (kg/m^2^)	24.4 (22.0–26.6)	26.9 (24.1–29.4)
BMI Category (kg/m^2^) Underweight, < 18.5 (%, n)	0% (0)	9% (1)
Normal weight, 18.5–24.9 (%, n)	56% (14)	18% (2)
Overweight, 25–30 (%, n)	24% (8)	55% (6)
Obese, > 30 (%, n)	12% (3)	18% (2)
Fat Mass Index (kg/m^2^)	2.7 (2.3–3.7)	3.8 (2.4–5.2)
Waist circumference (cm)	93 (88–100)	99 (89–110)
Proportion with increased (high-risk) sex-specific weight circumference (%increased, n)	40% (10)	55% (6)
*Physical activity and exercise*		
Total physical activity (MET-hrs/wk)	**19.2 (16.1–26.7)**	**11.1 (4.0–13.0)**
Intentional exercise (MET-hrs/wk)	5.9 (2.2–12.2)	2.8 (0.0–5.3)
Light-intensity exercise (MET-hrs/wk)	**11.1 (7.0–14.9)**	**3.5 (0.8–6.9)**
Moderate-intensity exercise (MET-hrs/wk)	3.6 (2.1–7.7)	1.3 (0.0–6.0)
Heavy-intensity exercise (MET-hrs/wk)	**1.3 (0.0–6.9)**	**0.0 (0.0–0.0)**

Means ± standard deviations are shown for normally distributed variables, and medians ± interquartile intervals are shown non-normally distributed variables. Normality of distribution was assessed using a Shapiro–Wilk test. Values in bold text indicate a statistically significant difference (*p* < 0.05) between individuals with FRDA versus healthy volunteers by Student’s *t* test or Kruskal–Wallis test, as appropriate. The number of GAA repeats in the FXN gene on the least affected allele correlates with earlier age of onset, more rapid disease progression, and severity of symptoms, but does not account for all phenotypic variability [1]

**Table 2:** Adult CrCEST MRI results, univariate analyses

	Healthy controls (*n* = 22)	Friedreich’s ataxia (*n* = 10)

Resting CrCEST (%asymmetry, index of free Cr concentration)		
LG	6.2 (5.3–8.2)	5.9 (4.9–6.4)
MG	5.9 (5.4–7.5)	6.3 (5.7–7.0)
Sol	6.6 (6.0–7.0)	7.0 (6.1–7.4)
ΔCrCEST with exercise (%asymmetry)		
LG	8.9 (6.0–10.4)	5.2 (2.9–9.1)
MG	5.0 (4.5–5.8)	3.5 (1.2–5.5)
Sol	3.4 (2.8–4.7)	3.9 (1.7–6.6)
τCr (seconds, index of OXPHOS capacity)		
LG	**138 (85–226)**	**274(221–309)**
MG	184 (90–263)	269 (240–342)
Sol	254(184–309)	210(149–266)

Medians ± interquartile intervals are shown for non-normally distributed variables. Normality of distribution was assessed using a Shapiro-Wilk test. Values in bold text indicate a statistically significant difference (*p* < 0.05) between individuals with FRDA versus healthy volunteers by Kruskal-Wallis test. Total participant numbers reflect that 3 participant scans (1 case and 2 controls) were excluded due to technical limitations, including motion artifacts, shimming and other image acquisition errors. TCr values less than one scan time (24 s) were excluded, including two observations in the MG

LG: lateral gastrocnemius, MG: medial gastrocnemius, Sol: soleus

**Table 3:** Adults with FRDA vs. healthy controls, mixed-effects regression model of resting CrCEST (%asymmetry, an index of free creatine concentration)

	Model 1 β, [CI]	Model 2 β, [CI]

Age (years)	0.005 [−0.03, 0.03]	−0.01 [−0.04, 0.02]
Male sex (vs. female)	−0.85 [−1.8, 0.07]	−1.1* [−1.9, −0.27]
FRDA (vs. no FRDA)	−0.27 [−1.1, 0.62]	0.13 [−0.67, 0.93]
BMI (kg/m^2^)	–	−0.11** [−0.19, −0.043]
Muscle Group		
LG (reference)	–	–
MG	0.07 [−0.23, 0.38]	0.07 [−0.23, 0.38]
Soleus	0.50** [0.19, 0.81]	0.50** [0.19, 0.81]

In each model, subject is included as a random effect and clinical covariates as fixed effects. The following fixed effects were included: Model 1, age, sex, disease status, muscle group; Model 2, age, sex, disease status, BMI, and muscle group. For both models, *n* = 32 participants contributed a total of *n* = 96 observations. No observations were excluded. β coefficient values are shown, with corresponding 95% confidence intervals. Results in bold text indicate statistically significant β coefficient values

* *p* < 0.05

** *p* < 0.01

*** *p* < 0.001

LG: lateral gastrocnemius, MG: medial gastrocnemius, Sol: soleus

**Table 4:** Adults with FRDA vs. healthy controls, linear regression model of ΔCrCEST with exercise (%asymmetry, an index of free creatine concentration) in the lateral gastrocnemius, where a larger ΔCrCEST represents a larger change with exercise

Covariate	Model 1 β [CI]	Model 2 β [CI]	Model 3 β [CI]

Age (years)	0.03 [−0.09, 0.16]	−0.04 [−0.16, 0.09]	0.02 [−0.09, 0.14]
Male sex (vs. female)	1.6 [−1.8, 5.0]	0.7 [−2.5, 3.0]	1.1 [−2.2, 4.4]
FRDA (vs. no FRDA)	−2.8 [−6.1, 0.4]	−0.47 [−4.1, 3.1]	−1.8 [−5.1, 1.5]
Total Physical Activity (MET-hrs/wk)	–	0.20* [0.03, 0.37]	–
Waist Circumference (cm)	–	–	−0.09 [−0.19, 0.01]

For all models, *n* = 32 participants contributed a total of *n* = 32 observations. No observations were excluded. The following covariates were included: Model 1, age, sex, and disease status (no additional covariates); Model 2, age, sex, disease status, and total physical activity; Model 3, age, sex, disease status, and waist circumference. β coefficient values are shown, with corresponding confidence intervals. Results in bold text indicate statistically significant β coefficient values

* *p* < 0.05

** *p* < 0.01

*** *p* < 0.001

**Table 5:** Adults with FRDA vs. healthy controls, linear regression models of post-exercise decline in CrCEST (τCr, in seconds) in the lateral gastrocnemius, where prolonged τCr suggests decreased OXPHOS capacity

Covariate	Model 1 β [CI]	Model 2 β [CI]	Model 3 β [CI]

Age (years)	2 [−2, 6]	− 1 [−5, 3]	3.9 [0, 8]
Male sex (vs. female)	86 [−21, 194]	91 [−25, 208]	107 [0, 215]*
FRDA (vs. no FRDA)	131* [28, 234]	131* **[8, 253]**	76 [−45, 196]
ΔCrCEST w/ Exercise	–	0 [−15, 15]	–
Resting CrCEST	–	− 1 [−43, 41]	–
Total Physical Activity (MET-hrs/wk)	–	–	− 5 [−10, 1]

For all models, *n* = 32 participants contributed a total of *n* = 32 observations. The following covariates were included: Model 1, age, sex, and disease status (no additional covariates); Model 2, age, sex, disease status, ΔCrCEST, and resting CrCEST; Model 3, age, sex, disease status, and total physical activity. β values are shown, with corresponding 95% confidence intervals. Results in bold text indicate statistically significant β coefficient values

* *p* < 0.05

** *p* < 0.01

*** *p* < 0.001

## Abbreviations

3 T: 3 Tesla
BMI: Body mass index
Cr: Creatine
CrCEST: Creatine chemical exchange saturation transfer
ΔCrCEST: Change in CrCEST with exercise
DXA: Dual-energy X-ray absorptiometry
FRDA: Friedreich’s ataxia
LG: Lateral gastrocnemius
MG: Medial gastrocnemius
MRI: Magnetic resonance imaging
MRS: Magnetic resonance spectroscopy
MTR_asym_: Magnetization transfer asymmetry
OXPHOS: Oxidative phosphorylation
PCr: Phosphocreatine
Sol: Soleus
τCr: Post-exercise CrCEST recovery exponential time constant
