# Supplementary material for: In vivo assessment of OXPHOS capacity using 3 T CrCEST MRI in Friedreich’s ataxia
Source: J Neurol. 2021 Oct 15;269(5):2527–38. doi: 10.1007/s00415-021-10821-1 (PMC9010488; doi:10.1007/s00415-021-10821-1)
Supplement: Supplementary file 1 — Supplementary file1 (PDF 191 KB) [file 415_2021_10821_MOESM1_ESM.pdf]

Supplemental Materials

Title: *In Vivo* Assessment of OXPHOS Capacity using 3T CrCEST MRI in Friedreich's Ataxia

Authors: Gayatri Maria Schur[1,2], Julia Dunn[1], Sara Nguyen[1], Anna Dedio[1], Kristin Wade, MA[1], Jaclyn Tamaroff, MD[1], Nithya Mitta[1], Neil Wilson, PhD[3], Ravinder Reddy, PhD[3], David R. Lynch, MD, PhD[4], Shana E. McCormack, MD, MTR[1,5]

Affiliations:

[1]Division of Endocrinology and Diabetes, The Children's Hospital of Philadelphia, Philadelphia, PA, 19104;

[2]Medical Scientist Training Program, New York University Grossman School of Medicine, New York, NY, 10016**;**

[3]Center for Magnetic Resonance and Optical Imaging, Department of Radiology, University of Pennsylvania, Philadelphia, PA, 19104;

[4]Division of Neurology, The Children's Hospital of Philadelphia, Philadelphia, PA, 19104;

[5]Department of Pediatrics, Perelman School of Medicine, University of Pennsylvania, Philadelphia, PA, 19104.

Address for Correspondence:

Gayatri Maria Schur

New York University Grossman School of Medicine

Vilcek Institute of Graduate Biomedical Sciences

550 First Avenue, MSB 228

New York, NY 10016

gayatri.schur@nyulangone.org

Supplementary Table 1. Adults with FRDA vs. healthy controls, linear regression model of ΔCrCEST with exercise (%asymmetry, an index of free creatine concentration), in the medial gastrocnemius and soleus, where a larger ΔCrCEST represents a larger change with exercise.

	**Medial Gastrocnemius**			**Soleus**		
**Covariate**	Model 1 β [CI]	Model 2 β [CI]	Model 3 β [CI]	Model 1 β [CI]	Model 2 β [CI]	Model 3 β [CI]
**Age (years)**	0.04 [-0.03, 0.11]	0.02 [-0.05, 0.10]	0.04 [-0.03, 0.10]	0.03 [-0.03, 0.10]	0.02 [-0.06, 0.09]	0.03 [-0.03, 0.09]
**Male sex (vs. female)**	0.08 [-1.78, 1.93]	-0.15 [-2.07, 1.76]	-0.25 [-2.01, 1.52]	1.66 [-0.16, 3.47]	1.43 [-0.44, 3.31]	1.32 [-0.39, 3.03]
**FRDA (vs. no FRDA)**	-1.6 [-3.4, 0.14]	-1.0 [-3.2, 1.1]	-1.0 [-2.8, 0.8]	0.45 [-1.3, 2.2]	1.35 [-1.1, 3.1]	1.0 [-1.1, 3.1]
**Total Physical Activity (MET-hrs/wk)**	-	0.05 [-0.05, 0.15]	-	-	0.05 [-0.05, 0.15]	-
**Waist Circumference (cm)**	-	-	**-0.06* [-0.1, -0.01]**	-	-	**-0.06* [-0.1, -0.01]**

For all models, n=32 participants contributed a total of n=32 observations. The following covariates were included: Model 1, age, sex, disease status (no additional covariates); Model 2, age, sex, disease status, and total physical activity; Model 3, age, sex, disease status, and waist circumference. β coefficient values are shown, with corresponding confidence intervals. Results in bold text indicate statistically significant β coefficient values: *p<0.05; **p<0.01; ***p<0.001.

Supplementary Table 2. Adults with FRDA vs. healthy controls, linear regression models of post-exercise decline in CrCEST (τCr, in seconds), in the medial gastrocnemius and soleus, where prolonged τCr suggests decreased OXPHOS capacity.

	**Medial Gastrocnemius**			**Soleus**		
**Covariate**	Model 1 β [CI]	Model 2 β [CI]	Model 3 β [CI]	Model 1 β [CI]	Model 2 β [CI]	Model 3 β [CI]
**Age (years)**	-1.1 [-5.4, 3.2]	-1.4 [-5.7, 2.9]	1.3 [-3.2, 5.9]	2.2 [-0.4, 4.7]	1.6 [-0.7, 4.0]	2.3 [-0.7, 5.2]
**Male sex (vs. female)**	91 [-25, 208]	46 [-75, 166]	**130 [17, 244]***	3.2 [-67, 73]	-38 [-108, 32]	4.9 [-69, 78]
**FRDA Disease Status**	52 [-62, 166]	51 [-65, 167]	-22 [-147, 103]	-30 [-97, 37]	-38 [-99, 23]	-34 [-117, 48]
**ΔCrCEST w/ Exercise**	-	7 [-18, 32]	-	-	**14* [0, 28]**	-
**Resting CrCEST**	-	-39 [-75, 3]	-	-	-18 [-44, 32]	-
**Total Physical Activity (MET-hrs/wk)**	-	-	**-7* [-13, 1]**	-	-	0 [-4, 4]

For medial gastrocnemius, n=30 participants contributed a total of n=30 observations. Two observations in the MG were less than one scan time (24s) excluded. For soleus, n=32 participants contributed a total of n=32 observations. The following covariates were included: Model 1, disease status, sex, and age (no additional covariates); Model 2, disease status, sex, age, ΔCrCEST, and resting CrCEST; Model 3, disease status, sex, age, and total physical activity. No statistically significant effects of sex and age were detected, results not shown. Beta values are shown, with corresponding 95% confidence intervals. Results in bold text indicate statistically significant beta coefficient values: *p<0.05; **p<0.01; *** p<0.001.

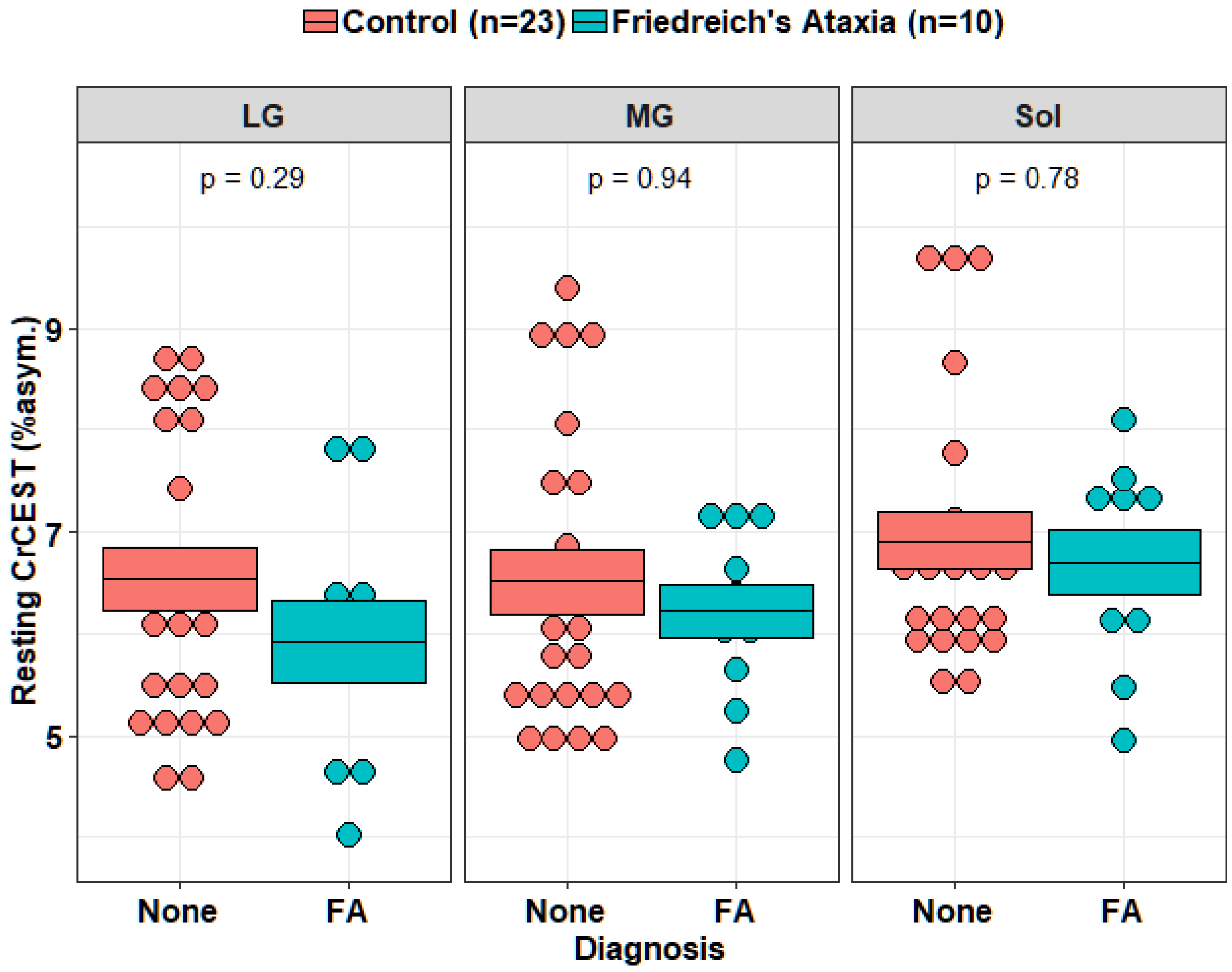

Supplementary Figure 1. Resting CrCEST (%asymmetry, an index of free creatine concentration) in adults. Orange corresponds to controls, and blue corresponds to adults with FRDA. p-values for differences by Kruskal-Wallis test are shown. LG: lateral gastrocnemius, MG: medial gastrocnemius, Sol: soleus.

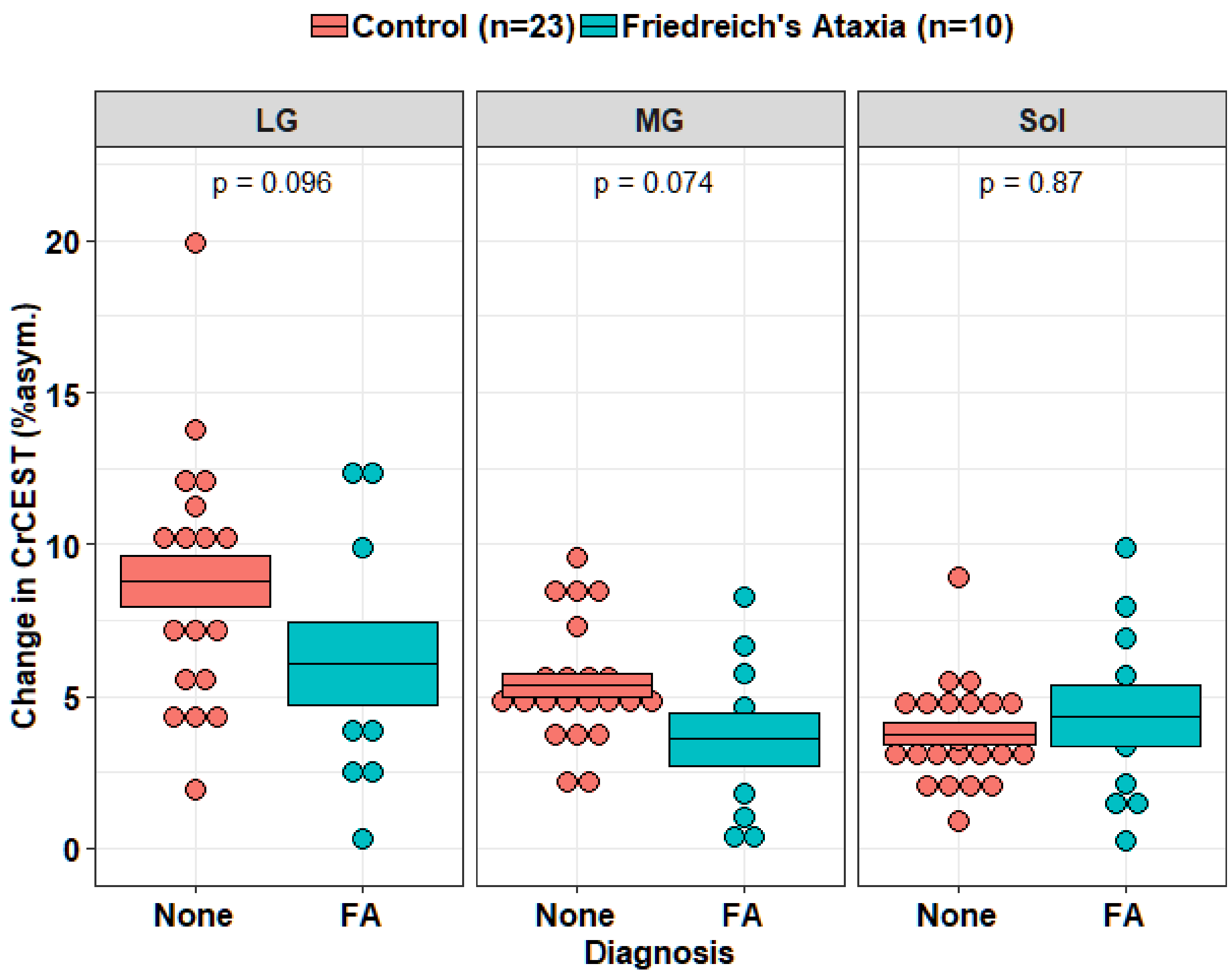

Supplementary Figure 2. ΔCrCEST with exercise (%asymmetry, an index of free creatine concentration) in adults. Orange corresponds to controls, and blue corresponds to adults with FRDA. P-values for differences by Kruskal-Wallis test are shown. LG: lateral gastrocnemius, MG: medial gastrocnemius, Sol: soleus.
